# Experts’ Needs Assessment and Evaluation of Mobile Apps for Children of Parents With Mental Illness: Mixed Methods Study

**DOI:** 10.2196/102905

**Published:** 2026-10-09

**Authors:** Victoria Lönnfjord, Leonardo Horn Iwaya, Charlotte Bäccman, Gisela Priebe

**Affiliations:** 1 Department of Social and Psychological Studies Centre for Research on the Mental Health and Life Circumstances of Children and Youth Karlstad University Karlstad, Värmland Sweden; 2 Department of Mathematics and Computer Science Karlstad University Karlstad, Värmland Sweden; 3 Department of Social and Psychological Studies CTF Service Research Center Karlstad University Karlstad, Värmland Sweden

**Keywords:** alcoholism, child of impaired parents, children of parents with mental illness, COPMI, digital health, empirical research, health promotion, mental health, mHealth, mobile apps, mobile health, substance abuse

## Abstract

**Background:**

Children of parents with mental health illness (COPMI) have the right to receive preventive interventions to avoid developing their own mental health and socioeconomic problems. However, access to interventions varies widely within health care and social services depending on their geographical location in Sweden. In this regard, mobile health (mHealth) interventions for COPMI provide a pathway for more equitable and sustainable preventive support.

**Objective:**

This project aims to (1) map and assess the quality of available mHealth apps relevant to COPMI; (2) investigate experts’ perceptions of COPMI needs for digital health solutions (particularly mHealth) by consulting and collaborating with an interdisciplinary reference group (scholars, child rights organizations, and IT professionals); and (3) explore the prerequisites for development, implementation, and sustainable access to future digital solutions for COPMI.

**Methods:**

We collaborated with a reference group through a series of meetings and workshops, identifying and assessing the quality of 9 free, highly ranked apps (ie, using the well-known Mobile App Rating Scale [MARS]). All the workshop sessions were recorded and transcribed for further qualitative analysis, allowing us to document and derive our main findings.

**Results:**

Three out of 9 apps received high scores across the MARS dimensions of aesthetics, perceived functionality, information, and engagement in the expert heuristic evaluation using recorded demonstrations of the apps, indicating a significant lack of high-quality apps relevant to COPMI. Further findings were derived, such as the experts’ preference for more general apps that are not specifically targeted to COPMI, as these could promote self-identification and reduce stigmatization. Regarding the third aim, the results showed that it was important to find an app that protected users’ privacy by allowing anonymous access to digital support and that mobile apps should be complemented (or replaced) by web-based applications to improve accessibility for children who may not be allowed to download apps without parental permission.

**Conclusions:**

The sustainability of digital solutions (web applications or mobile apps) for COPMI is the biggest challenge for future developments. Long-term impact requires stable funding, continuous maintenance, and partnering with established organizations that already possess the infrastructure, expertise, and reach necessary to provide ongoing support. To address the limitations in the study design and deployment issues, it is important to actively involve children through participatory and co-creational approaches when designing and developing mHealth solutions. Also, future evaluations should include privacy and data security assessments such as app permissions, privacy-policy presence, and third-party sharing.

## Introduction

### Overview

About 11% of all children born in Sweden during 1991 and 2011 had a parent with a mental illness treated within secondary care [1]. A scoping review of international research shows that 10% to 20% of all children and youth grow up with a parent with severe mental illness [2]. These children have up to a 50% risk of developing a mental illness, with some variations depending on the type of illness [3]. Children of parents with mental illness (COPMI) also have a markedly increased risk of living with broad socioeconomic adversity (eg, public assistance and lowest income quintile) than do other children [1]. Health-economic calculations have shown a large amount of long-term additional costs for COPMI when they grow up, related to differences in mental illness, including alcohol or drug abuse, compared with children who grow up in families without these challenges [4].

Preventive interventions may decrease the risk of problem development by 40% [3]. In a systematic review and meta-analysis, Moltrecht et al [5] found 30 different interventions, often with small or mixed effects on, for example, child problems, parent symptoms, or parenting. Some of these methods have been implemented and evaluated in Sweden [6,7]. The Swedish Health and Medical Services Act (HSL; SFS 2017:30) has required since 2010 that COPMI be identified and offered information, counseling, and support. Despite this requirement, only a small proportion of these children receive the services they need, and collaboration between health care and social services remains underdeveloped [8].

The availability and type of support regarding health care and social services vary considerably depending on where in Sweden COPMI and their families live. Welfare systems in Sweden often focus on the individual rather than the family, resulting in low prioritization of support for COPMI [9], especially in times of scarce resources. Parents with mental health problems or substance abuse disorders may also struggle with stigma and shame, which can make them hesitant to accept support for their children [10]. This is in line with family systems theory [11], which emphasizes how family patterns of interaction shape daily life. These patterns will also affect how these families engage with external systems such as schools, social services, and health care providers.

In families with parental mental illness, including alcohol or substance abuse, there may be a reversal of family structure, in which the child takes on responsibilities that are normally the responsibility of a parent. Emotional instability and unpredictability may also negatively affect the child’s sense of safety, trust, and coherence. Children and youth who take on parental responsibilities are called young carers [12]. In addition, not all parents with mental illness are in contact with health care or social services, which makes it challenging to identify the children. Studies with children show that they want information about their parents’ difficulties, delivered in a tailored and age-appropriate manner [13].

With that in mind, digital health technologies, particularly mobile health (mHealth) apps, have been shown to significantly improve access to psychological support. A recent umbrella review on mental health apps by Koh et al [14] highlights their potential to provide timely support, reduce mental health care costs, combat stigma in help-seeking, and improve therapeutic outcomes. However, they also identified significant pitfalls related to user engagement, safety in emergency situations, privacy and confidentiality breaches, and the use of non–evidence-based approaches. Although risks and benefits should be balanced, COPMI’s unmet psychological support needs to be addressed, and mHealth apps can be crucial to ensuring equitable and accessible support in underserved regions of the country.

mHealth apps are being increasingly used by both young and older adult populations, empowering individuals to take better care of themselves by enhancing their autonomy and self-efficacy regarding their mental health [15,16]. mHealth apps can provide continuous, real-time support in everyday settings, facilitate emotional self-monitoring, and promote coping strategies, which may be particularly relevant for COPMI who may not seek formal help [17]. They also offer advantages such as accessibility, anonymity, and reduced stigma, which can increase engagement with mental health support [18].

Emerging evidence further suggests that mHealth interventions can support well-being and self-regulation, for example, through mindfulness-based approaches, although the quality and effectiveness of available apps vary widely and remain insufficiently evaluated [19]. Adolescents generally engage well with mobile mental health tools and perceive them as useful, particularly for increasing emotional awareness and supporting self-help [17,18]. However, despite the rapid growth in available apps, many lack an evidence base, and guidance on their quality and suitability for specific vulnerable groups remains limited [18,19].

Importantly, research on mHealth app development highlights that effectiveness depends not only on accessibility but also on key design features. Apps that incorporate behavior change principles—such as motivation, self-efficacy, personalized feedback, and social support—are more likely to be engaging and effective in supporting positive outcomes [20]. Conversely, mHealth apps that lack theoretical grounding, personal relevance, or usability often fail to sustain engagement. This underscores the importance of developing and evaluating digital interventions that are both evidence-based and tailored to users’ needs.

Taken together, these findings highlight the potential of mobile apps as scalable tools for supporting COPMI, while also pointing to critical knowledge gaps regarding their feasibility, acceptability, and usefulness for this specific group. There is therefore a clear need to evaluate and further develop digital interventions grounded in evidence and adapted to COPMI’s needs and contexts.

In summary, COPMI have the right to receive support and preventive interventions to strengthen their mental well-being and prevent their own mental health issues and socioeconomic problems. However, access to interventions varies widely within Sweden’s health care and social services. This is why we have collaborated with well-established and well-known child rights organizations with the potential to reach out to COPMI with digital interventions on a national level. The aim is not to replace but to complement public health or social service interventions for more equal and health-promoting health care by leveraging digital health solutions.

### Purpose, Aims, Collaboration, and Conditions

The purpose of this research project was to contribute to equitable and sustainable preventive digital health interventions (mobile phone–based or web-based) for COPMI. We started with this exploratory mixed methods study with 3 specific aims:

- Aim 1: the aim was to map and assess the quality of available mHealth apps relevant to COPMI.
- Aim 2: the aim was to investigate experts’ perceptions of COPMI needs for digital health solutions, particularly mHealth, through consultation and collaboration with an interdisciplinary reference group (RG) comprising academic researchers, child rights organizations, and IT professionals.
- Aim 3: the aim was to explore the prerequisites for the development, evaluation, implementation, sustainable access, and future development of digital solutions for COPMI.

Special attention was paid to exploring prerequisites for implementing digital interventions in the long run, following up on this research study. Our research includes close collaboration with an RG with professionals and researchers from academia, the public sector, private sector, and child rights organizations.

## Methods

### Overview

This research project was conducted from February 2025 to January 2026. The research activities included a series of workshops involving a diverse RG and the identification and evaluation of mobile apps relevant to our target population. A summary of all the activities is shown in Figure 1, which is further detailed in the following subsections.

**Figure 1:**
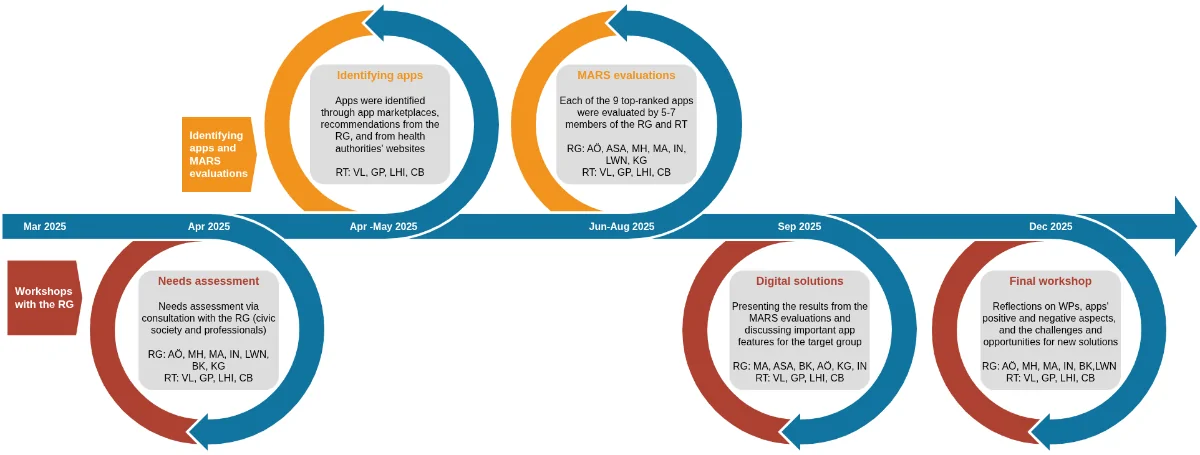
Overview of the project’s main activities, including workshops, app identification, and Mobile App Rating Scale (MARS) evaluation, and description of participating experts and researchers. We use the initials of the researchers (ie, research team [RT]) and the experts (ie, reference group [RG]) to indicate which activities each person participated in. AÖ: Anneli Öhrling; ASA: Ala Sarah Alaqra; BK: Bridget Kane; CB: Charlotte Bäccman; GP: Gisela Priebe; IN: Ida Nilsson; KG: Klara Gustavsson; LHI: Leonardo Horn Iwaya; LWN: Lina Wirehag Nordh; MA: Maria Afzelius; MH: Monika Högsnes; VL: Victoria Lönnfjord.

### The Project’s Research Team and RG

We refer to the authors and core members of the research project as the research team (RT). During the project, we collaborated with an RG that was made up of 8 experts. In brief, the RG included 5 academics from different universities (Karlstad University, University of Gothenburg, and Malmö University), 3 of whom also have clinical experience working with COPMI; 2 practitioners from nonprofit child rights organizations (BRIS and Maskrosbarn); and 1 developer from a software company (StickyBeat).

The experts from the child rights organizations and those with clinical experience were especially important, as they shared their firsthand professional experience working with COPMI, helping us to better understand our target population’s needs for digital support. The RT contributed expertise from the areas of psychology, social sciences, computer science, and digital health.

We also received support from a mobile developer from a software company, which helped us understand the practicalities of designing and deploying mobile apps. Nonetheless, both the RT and RG were continuously engaged and took part in several activities, such as workshops, project meetings, and mobile app evaluations.

### Workshops for Needs Assessment, Digital Solutions, and Prerequisites for Digital Solutions

Digital workshops were the main method adopted for the experts’ needs assessments of digital solutions for COPMI and were directly connected to aim 2. Workshops are used to acquire new knowledge, solve problems, or create innovation concerning a topic, producing reliable and valid data that could benefit, for example, organizational change and design [21]. As a methodology, previous research [22] found them useful in identifying, articulating, and exploring challenges in relation to research involving technology.

Activities with the RG included a kick-off meeting prior to the workshops, where we explained the whole project and got better acquainted. The RG also participated in 3 workshops, with the first workshop (WS1) focusing on the COPMI needs assessment. The second workshop (WS2) focused on the RG’s ideas for a digital solution (eg, particularly highly personalized ones, such as mobile apps) and factors related to the sustainability of digital health solutions (eg, organizational factors, maintenance of the system, and system providers/financers). The third workshop (WS3) focused on presenting the study’s results to the RG and discussing challenges and opportunities in the use of digital solutions, especially mobile apps.

As shown in Figure 1, the RT took part in all workshops, with a varying number of RG members joining each of them (ie, n=7 in WS1; n=6 in WS2; and n=6 in WS3). Each workshop lasted 3 hours and was held digitally via Zoom (Zoom Communications Inc) to facilitate participation, and we also split the group into breakout rooms to facilitate discussions. We also used the Miro Board [23], allowing participants to write digital “Post-It” notes used for brainstorming, organizing ideas, taking notes, and collaborating in real time.

The data from the workshops were recorded, transcribed, and analyzed using qualitative content analysis [24], focusing on the manifested content of the respondents’ statements, in other words, what was explicitly expressed during the workshops. The researchers first familiarized themselves with the material by reading through the transcripts and the information obtained from the Miro Boards. We used NVivo (Lumivero) to code the material. In the first step, the material was coded separately following each workshop. Meaning units were identified and condensed. In the second step, the meaning units were coded and analyzed to identify patterns. These results were then presented at the following workshop; hence, results from WS1 were presented at WS2, and results from WS2 were presented at WS3, creating an iterative feedback loop to the RG. Since the workshops had different focuses (needs assessment, digital solution, and prerequisites for digital solutions) and results from previous workshops were discussed, a final step was taken in the analysis. After coding WS3, the codes for the whole project were revisited to find patterns that gave the full description of the different parts of the result, which is connected to aim 3.

### Identifying and Assessing Mobile Apps for COPMI

As shown in Figure 1, parallel to fulfilling aim 1, we also addressed aim 2 of our project on the mapping and interdisciplinary quality assessment of available apps. In this activity, we sought to identify free and highly ranked apps in English or Swedish on the market that could be relevant to our target group. Figure 2 summarizes the steps of the app search process. To identify the apps, we consulted websites from public health care institutions that had lists of recommended apps for youth support, including NHS Dorset [25], NHS Berkshire Healthcare [26], University College Dublin [27], and CAMHS [28], resulting in a list of 80 apps. Besides that, we also included 10 apps suggested by the RG, for example, apps that they were aware of or had previously used. These lists were reviewed by the entire RT, and after removing the duplicates, we identified 65 relevant apps.

**Figure 2:**
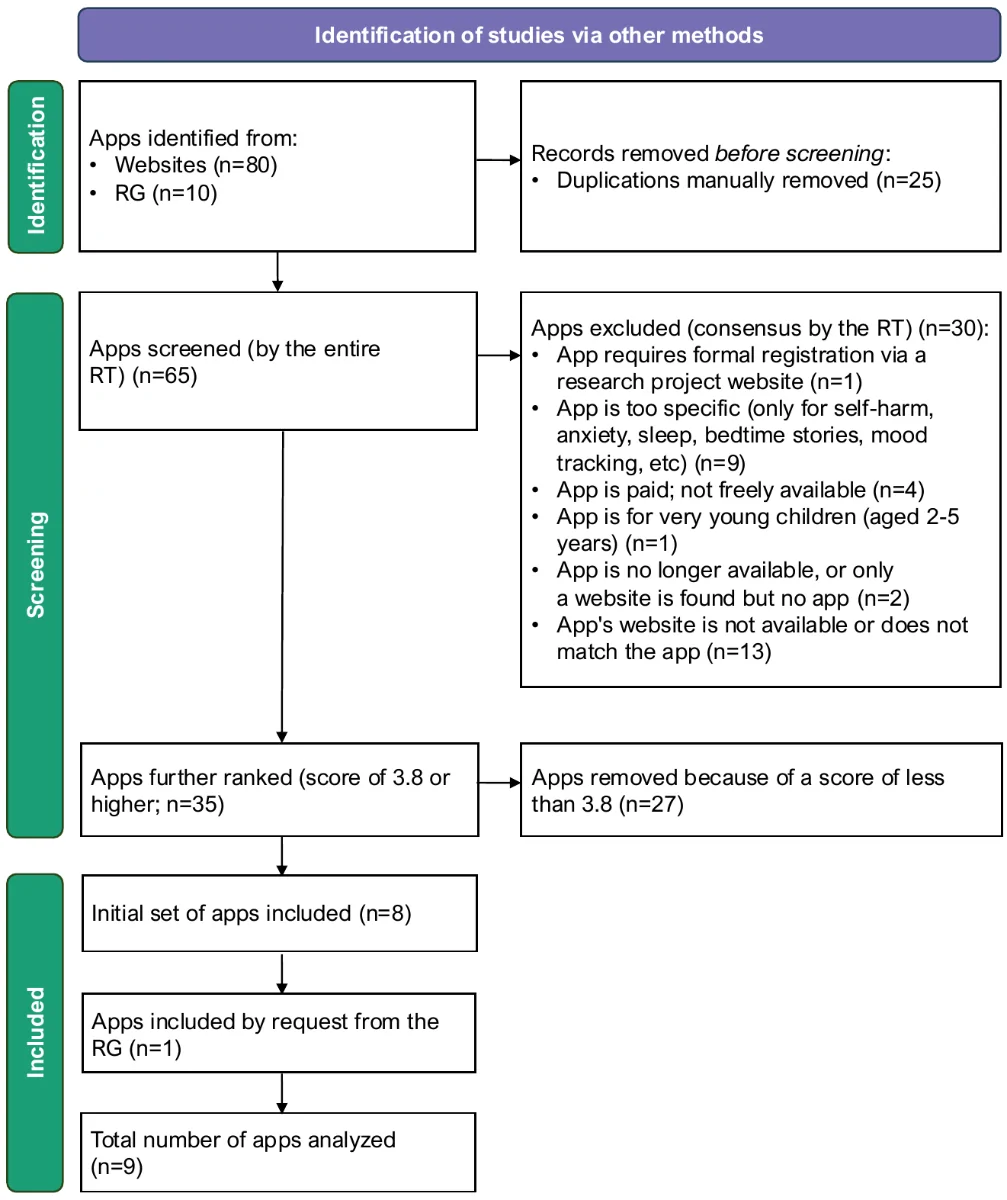
Flowchart with a summary of the search process for identifying mobile apps relevant for children of parents with mental health illness (conducted from April to May 2025 by the research team [RT] and reference group [RG]).

After identifying 35 relevant apps, we further shortlisted them based on their 5-star ratings on Google Play Store, including only apps with a score of 3.8 or more (ie, highly ranked). Eight apps were shortlisted, including “Safe Place” [29] and “Snorkelövning” (available in English and Swedish), and “Combined Minds,” “FearTools - Anxiety Aid,” “MoodTools - Depression Aid,” “Smiling Mind: Mental Wellbeing,” “Calm - Sleep, Meditate, Relax,” and “Insight Timer - Meditation App” (available only in English). After consulting with the RG, we also decided to include a more recently released app, “ME-WE young carers” (available in Swedish), as the RG and RT had an interest in having it also assessed. It is worth mentioning that the apps Safe Place and Snorkelövning were already known or used by some members of the RG. In total, 9 apps were included in the study to be further assessed, as provided in Table 1.

**Table 1:** Overview of the apps included in the study, showing the app name, unique app identifier (appId), number of installs, and score (Google Play Store data from April 2025).

App	appId	Installs, n	Score
Calm - Sleep, Meditate, Relax	com.calm.android	62,517,010	4.4
Insight Timer - Meditation App	com.spotlightsix.zentimerlite2	101,35,138	4.7
Smiling Mind: Mental Wellbeing	com.smilingmind.app	1,555,031	4.3
MoodTools - Depression Aid	com.moodtools.moodtools	381,740	4.1
FearTools - Anxiety Aid	com.feartools.feartools	82,126	4.4
Safe Place	com.raddabarnen.calm	41,926	4.6
Snorkelövning	se.lul.snorkel	18,763	3.8
Combined Minds	uk.org.stem4.combinedminds	15,433	3.8
ME-WE young carers	se.appbolaget.mewe	189	N/A^a^

^a^N/A: not applicable.

After identifying and selecting the best available apps, 5 to 7 members of the RT and RG performed a quality assessment using the well-known Mobile App Rating Scale (MARS) [30]. MARS guides the evaluation of the apps across 4 main dimensions (ie, engagement, functionality, aesthetics, and information quality), allowing us to rank the top apps that could be adopted by the target group or used as a baseline for a future pilot project or digital intervention.

All members of the RT and RG received basic training on MARS, explaining the rationale behind the scale and its dimensions and going over a practical assessment of a mobile app used as an example. This training consisted of a prerecorded video explaining and exemplifying MARS, followed by a dedicated online session to discuss, ask questions, or clarify any doubts. The training materials and session were organized by LHI (from the RT), who already had previous experience working with MARS as well as expertise in usability research.

To facilitate the MARS evaluations, we used recordings of the apps, in which a researcher (LHI) went over all the main functionalities of the app, describing all the steps taken and explaining the elements and features (ie, a “think-aloud” method), with app recordings lasting 21 minutes on average. Fake credentials were used (ie, a fake email address and username) to create our accounts in each app, avoiding the use of the RT’s and RG’s personal information. After watching an app recording, the RT and RG members used a dedicated web form (ie, Google Forms) to provide their answers to the MARS questionnaire for that app, automatically storing their evaluations in a final spreadsheet (ie, Google Sheets). This way, it was easier for the RT to compile a final list of all evaluations for all the apps and quickly export the results for further analysis. The MARS evaluation took place during June and August 2025.

Several reasons led us to rely on app recordings, such as the fact that members of the RT and RG did not need to create new accounts for each of the apps that they evaluated, while also avoiding problems with app installation on their personal mobile devices. Some also did not have access to an Android device for the app evaluations. The members of the RG also had rather limited availability, so there was a need to reduce the time overhead imposed on them. In this way, the app recordings were crucial in this project to enable as many members of the RT and RG as possible to evaluate apps efficiently and independently, that is, scaling the number of evaluations per app. Because the evaluations were conducted using narrated screen recordings, rather than direct interactions with the apps, the MARS scores should be interpreted as heuristic expert evaluations of app interfaces, content, and demonstrated functionality. Hence, the scores do not represent actual user experience or engagement with the apps.

Apart from using MARS, the psychology researchers in the RT (CB and GP) also reviewed the content provided by the apps to validate the quality of the information they provide (ie, accurate and evidence-based content).

As a result, this activity supported us in compiling a list of the best apps that could be relevant to COPMI, detailing the types of features and functionalities that are most useful, the best engagement strategies used in the apps (eg, gamification, schedules, and reminders), and the best content and resources. This discussion on the evaluated apps and the use of MARS was part of WS2 on “digital solutions” (shown in Figure 1). Hence, these findings support the elicitation of requirements for leveraging existing or developing entirely new apps connected to the activities of aim 3.

Integration of quantitative and qualitative findings occurred during WS2 and WS3, where MARS results were discussed within the workshops and subsequently compared with themes from the qualitative analysis to generate a more comprehensive understanding of app quality, user needs, and implementation challenges.

### Project Public Repository

The public repository for research data and generated materials for this project is available at GitHub [31].

### Ethical Considerations

The project was reviewed by the University Ethics Advisor (HS 2025/228). The project had a suitable data plan and did not collect, process, or store sensitive personal information from the RG and RT during the workshops. The local Research Ethics Committee at Karlstad University assessed that the inclusion of professionals—people in the RG—would not fall under the act and hence would not require a full application to the National Research Ethics Committee. All participants in the RG received written and oral information about the study and were informed that they could withdraw from the study at any time. In the Results section, no data from specific individuals can be identified.

## Results

### Overview

Here, we present the main findings for the 3 project aims, starting with the results on the mapping and quality assessments of the apps (aim 1). This is followed by a narrative summary of the main topics (linked to aims 2 and 3), covering the needs assessment, exploration of prerequisites for developing, reflections on the MARS evaluations, and sustainable access to the apps by COPMI, as well as an integration of the mixed methods findings.

### Quantitative Results From the MARS Evaluations

As provided in Table 2, a total of 11 people (4 from the RT and 7 from the RG) took part in the MARS evaluations of the 9 apps selected in this study, with members from the RT evaluating all 9 apps and members from the RG evaluating 2 to 3 apps each.

**Table 2:** List of apps, their respective evaluators (displayed by initials), and the number of evaluators assigned to each app.

App	Number of evaluators	Evaluators
Calm - Sleep, Meditate, Relax	5	CB^a^, GP^b^, LHI^c^, MH^d^, VL^e^
Combined Minds	6	GP, LWN^f^, LHI^g^, MA^h^, BK^i^, VL
FearTools - Anxiety Aid	7	ASA^j^, AÖ^k^, CB, GP, IN, LHI, VL
Insight Timer - Meditation App	5	CB, GP, LHI, MH, VL
ME-WE young carers	6	CB, GP, IN, KG^l^, LHI, VL
MoodTools - Depression Aid	6	ASA, AÖ, CB, GP, LHI, VL
Safe Place	6	CB, GP, LWN, LHI, MA, VL
Smiling Mind: Mental Wellbeing	7	CB, GP, IN, KG, LHI, MH, VL
Snorkelövning	6	CB, GP, LWN, LHI, MA, VL

^a^CB: Charlotte Bäccman.

^b^GP: Gisela Priebe.

^c^LHI: Leonardo Horn Iwaya.

^d^MH: Monika Högsnes.

^e^VL: Victoria Lönnfjord.

^f^LWN: Lina Wirehag Nordh.

^g^LHI: Leonardo Horn Iwaya.

^h^MA: Maria Afzelius.

^i^BK: Bridget Kane.

^j^ASA: Ala Sarah Alaqra.

^k^AÖ: Anneli Öhrling.

^l^KG: Klara Gustavsson.

The average scores for each of the 5 sections of the MARS are shown in Table 3. Each section included 3 to 6 items. The range for the evaluation was 1 to 5, with high scores indicating good quality. We categorized the apps into 3 tiers:

- Low: FearTools - Anxiety Aid, ME-WE young carers, and MoodTools - Depression Aid, receiving the lowest scores (ie, <3.0)
- Medium: Calm - Sleep, Meditate, Relax; Combined Minds; and Snorkelövning in the middle (ie, >3.0 and <3.6)
- High: Insight Timer - Meditation App, Safe Place, and Smiling Mind: Mental Wellbeing, achieving the highest scores (ie, >3.8)

**Table 3:** Average scores for Mobile App Rating Scale sections, overall average score for each app, and overall average scores across apps for each section.

App	Engagement	Functionality	Aesthetics	Information	Subjective quality	Average
Safe Place	4.1	4.0	4.2	4.1	3.5	4.0
Insight Timer - Meditation App	3.9	3.9	4.3	3.7	3.3	3.8
Smiling Mind: Mental Wellbeing	3.6	3.8	4.1	3.8	3.4	3.8
Combined Minds	3.2	3.8	3.9	3.8	3.0	3.6
Snorkelövning	3.1	3.8	3.3	3.9	3.1	3.4
Calm - Sleep, Meditate, Relax	3.2	3.35	3.8	3.25	2.55	3.2
ME-WE young carers	2.1	3.3	2.8	3.3	1.9	2.7
FearTools - Anxiety Aid	2.4	3.4	2.4	3.0	1.8	2.6
MoodTools - Depression Aid	2.5	3.2	2.7	2.7	1.8	2.6
Mean across apps, mean (SD)	3.1 (0.65)	3.6 (0.29)	3.5 (0.68)	3.5 (0.44)	2.7 (0.67)	3.3 (0.52)

Considering the overall average scores, the apps tended to score highest under the MARS section of functionality (ie, the app functions well and does not crash, freeze, or have bugs; it is easy to learn, with easy navigation, flow logic, and gestural design). This suggests that, based on the recorded demonstrations, the evaluators perceived the apps as having generally clear navigation structures and demonstrated functionality. This was expected, as all the apps are rather simple software systems and should perform well on mobile devices.

However, the overall average for subjective quality (ie, willingness to recommend, use, or pay for the app) reveals the lowest scores, showing that for most apps, the evaluators indicated a limited willingness to recommend the apps to COPMI based on the recorded demonstrations, considering them to be of limited frequent use and with a below-average overall rating (ie, <3.0). The aspects of willingness to recommend an app and to use it frequently were the main factors, which particularly match the top-performing apps, Insight Timer - Meditation App, Safe Place, and Smiling Mind: Mental Wellbeing. Conversely, the worst-performing apps, FearTools - Anxiety Aid, ME-WE young carers, and MoodTools - Depression Aid, also received the lowest scores for subjective quality.

The overall averages for the engagement section (ie, the app is fun, interesting, customizable, interactive, and well-targeted to the audience) were the second lowest. Based on the recorded demonstrations, some apps were perceived as offering limited opportunities for engagement according to the evaluators, particularly true for low-scoring apps like FearTools - Anxiety Aid, ME-WE young carers, and MoodTools - Depression Aid. In terms of engagement, the top-performing apps were again Insight Timer - Meditation App, Safe Place, and Smiling Mind: Mental Wellbeing, according to the evaluators.

Most apps scored above average (ie, >3.0) under the aesthetics section (ie, good graphic design, overall visual appeal, color scheme, and stylistic consistency). Most apps scored well in this section due to their professional UI design and visual appeal, which is expected from top-ranked apps in the Android market. However, the lowest scores were given to FearTools - Anxiety Aid, ME-WE young carers, and MoodTools - Depression Aid, particularly owing to their low visual appeal. Performing exceptionally well under aesthetics were again the apps Insight Timer - Meditation App, Safe Place, and Smiling Mind: Mental Wellbeing, showcasing beautiful user interface design, graphic quality, and style consistency.

Finally, in terms of information (ie, the app contains high-quality information from credible sources), most apps performed reasonably well, achieving scores above 3.0. For this section, the evaluators were also checking that the information is adequate for the target group, for example, not excessive, not too complicated, and easy to understand. Many apps scored well (ie, >3.7) for this section, such as Combined Minds, Insight Timer - Meditation App, Safe Place, Smiling Mind: Mental Wellbeing, and Snorkelövning. Only the app MoodTools - Depression Aid scored relatively low (ie, 2.7), especially due to the limited visual explanations (ie, it uses mostly text and no charts, figures, or videos) and the lack of well-targeted content.

Under the information section, we also checked whether the apps have enough scientific evidence behind them (eg, having been trialed or tested in RCTs). We found enough evidence only for the apps Calm - Sleep, Meditate, Relax and Smiling Mind: Mental Wellbeing, which show several scientific articles on their websites, so it is easy to access and verify the scientific literature around them. Nonetheless, we also found studies on the use of the ME-WE young carers and MoodTools - Depression Aid apps, suggesting some level of scientific evidence. However, the majority of apps still lack scientific evidence on their use and efficacy, especially concerning children.

### Qualitative Results From the MARS Evaluations

When discussing the MARS evaluations during WS2, the RG expressed important aspects that they considered when rating the apps. One aspect was the level of user-friendliness. The app design, content, and features should also guide the user through the app. For example: “Does the user understand what to do in the app? What happens if I press this button?” Those aspects are important, especially when designing an app for children and youth. Furthermore, the app should have a supportive and caring approach, and it is important that it contains links to additional help and support. Another aspect mentioned as important when rating was whether the app was well-structured and had divided the content in a good way. How content is perceived and absorbed also felt important to consider; for example, too many features and settings are distracting. It is also important to bear in mind how a person feels mentally when using the app, which might affect the user’s experience.

Summarizing all the positive aspects mentioned in relation to the apps that the RG and RT rated, an app should have a stylish and aesthetically appealing design, and it should create a calm and secure feeling with a pedagogical and professional layout. The app should be easy to grasp and include just the right amount of content and good exercises. It is good if the app has personalized features and supportive elements (eg, sending emojis and links to help). Many in the RG appreciate it if the app can be used by different target groups, but also that it works well for specific groups, for example, COPMI. It is also seen as an advantage if it is available in Swedish and with an accompanying website.

Summarizing all the negative aspects mentioned in relation to the apps that the RG and RT rated, an app should not require framing (ie, explaining to a young person how or why they should use a certain app) and adult support to function properly. Another negative aspect is when it is unclear who the app is intended for. The content of an app can be correct and well-written; however, it may not be relevant to the user. A negative aspect is whether there is a paywall restricting access to all functions (eg, premium features). It is not good if an app has difficult-to-navigate menus, unclear structure, or incomprehensible or lengthy texts, which may cause users to “get lost” or find it hard to understand what to do. Furthermore, it is perceived as negative if the app has limited content and few features and/or exercises. There needs to be a variety of exercises and additional features. Furthermore, it is perceived as distracting if different narrators are used in different exercises.

### Integrated Mixed Methods Findings

As part of the mixed methods analysis, the quantitative MARS results were integrated with the qualitative workshop findings to explore areas of convergence and complementarity. The joint display presented in Table 4 illustrates how themes identified by the RG help explain patterns observed in the MARS assessment, particularly regarding engagement, subjective quality, and the suitability of existing apps for COPMI.

**Table 4:** Integration of quantitative MARS^a^ findings and qualitative workshop interpretations.

Quantitative MARS finding	Qualitative explanation from workshops	Integrated interpretation
Engagement received the second-lowest overall score (mean 3.1, SD 0.65). Low-performing apps such as ME-WE young carers, FearTools - Anxiety Aid, and MoodTools - Depression aid scored particularly low for engagement.	Workshop participants described poorly engaging apps as having limited content, few exercises, excessive text, unclear target audiences, and a need for adult framing. Participants emphasized personalization, supportive features, variety, and intuitive guidance as important for maintaining interest.	The low engagement scores from the heuristic evaluations may reflect limitations regarding personalization, limited interactivity, and insufficient tailoring to young users’ needs. Future COPMI^b^ solutions should prioritize co-design, personalization, and interactive features.
Functionality received the highest average score (mean 3.6, SD 0.29).	Participants highlighted the importance of intuitive navigation and clear structure when evaluating apps.	Perceived ease of use, as assessed in heuristic evaluations, appears necessary but not sufficient for sustained engagement. Good technical functionality alone seemed insufficient to generate strong overall impressions.
Subjective quality was the lowest-rated dimension (mean 2.7, SD 0.67), reflecting limited willingness to recommend or regularly use several apps.	Workshop discussions stressed the importance of relevance, supportiveness, meaningful content, and suitability for COPMI. Apps perceived as generic or poorly targeted were viewed negatively.	Participants’ reluctance to recommend apps may reflect a mismatch between the app content demonstrated and the psychosocial support needs identified for COPMI.
Information quality generally scored well (mean 3.5, SD 0.44).	The first workshop highlighted demand for tailored information about parental mental illness, children’s rights, coping strategies, and support options.	Existing apps provide reliable psychoeducative information. Yet, the apps may not adequately address the specific information needs that experts identified for COPMI.

^a^MARS: Mobile App Rating Scale.

^b^COPMI: children of parents with mental health illness.

### Results From the Needs Assessment

According to the needs assessment with the RG (aim 2), the COPMI needs identified by the RG support a service that considers the anonymity of the youth. It should be individualized, compensatory, provide positive role models, and encourage participation. The support should be psychosocial in nature and designed as a low-threshold service. In addition, it is important to provide meaningful activities. The support can be designed to either include support and care with parents, without parents, or for parents.

One aspect that the RG thinks is important to consider is the youth’s understanding of belonging to the target group. Youth need secure relationships and a general sense of safety, and not to feel alone in their situation. Hence, it is pivotal that youth have the opportunity to meet others in similar situations. Furthermore, it is important to develop a support network for COPMI.

The RG-identified COPMI needs are, for example, tailored knowledge, knowledge about action strategies, their rights, practical information, and information about COPMI in general. Both more general and specific digital solutions can be a complement to face-to-face support.

Many aspects of what the professionals thought were missing regarding support for COPMI aligned with the needs the group identified (eg, certain information and knowledge, opportunities to meet others in similar situations, and getting from insight to action in seeking support). In addition, the RG pointed out that it is important to ask young people if they want support and to listen to them. Youth often lack equitable access to support, so it is important to ensure that digital solutions are readily available for enhancing support. The support should be quality-assured, provided at the right time, and include different types of support. It should be designed to include various ways to share experiences (writing a diary, questions and answers, listening to others, etc). Specific content that the RG thinks is missing includes information about the parent’s care and involvement in the parent’s care, as well as content to reduce potential feelings of shame. Overall, there is a need to strengthen children’s trust.

The digital solutions need to include tailored support and multiple ways of delivering information, knowledge, and support, and they should be easy to find. They could also be designed from different perspectives (ie, children, parents, and professionals). Specific content or functions could include chatting forums, online training, shared stories and tips from others in similar situations, information and knowledge about the parent’s diagnosis or illness, information specific to the COPMI group, and support designed for or together with parents. The RG also repeatedly mentioned the need for data security and privacy requirements for such digital solutions.

### Challenges When Developing a Digital Solution

Connected to aim 3, the main challenge is the high cost to develop and maintain apps. It is also important to decide who is going to be the software owner, maintainer, and/or administrator. However, one should bear in mind that there could be different types of administrators, for example, one for technical and another for content-related aspects. Funding is also a challenge when developing digital solutions, especially if the development is funded in project form. In addition, the project form of funding entails problems in relation to long-term management, administrators, and updates. Another challenge mentioned by the RG refers to the app’s implementation aspects. These are aspects that need to be considered before the development of a digital solution starts.

Another challenge relates to reaching the target audience. There seems to be an overreliance on civil society and what they can accomplish when developing digital support for the target group. Nongovernmental organizations need resources and financing if they are going to take roles in relation to the development, maintenance, implementation, or other administrative tasks of digital solutions. Finally, challenges are related to aspects like parental control (eg, needing parental consent to buy or download apps) and security/privacy aspects. For example, it may be necessary to complement the app with web-based support, which does not necessitate parental consent for downloading an app. It is also important that the digital solution does not have any data leakage and respects children’s privacy.

Some solutions to the challenges brought up by the RG were to develop a plan for long-term maintenance. For example, digital companies may have maintenance agreements. Another aspect brought up by the RG was to learn from other similar projects, what worked and what did not, and for what reason. Since the RG was unanimous that the digital solution should be free and accessible to everyone and have no advertisements or commercials, aspects related to funding are crucial to solve. If the digital solution is (partly) funded by research grants, researchers could arrange education for a fee, and it could be in relation to the topic of the digital solution, maybe for other stakeholders with interests in COPMI (eg, professionals in need of education). Another solution is to team up with nongovernmental organizations or other stakeholders with interest in the target group. It is also essential to consider that it is cheaper to develop and maintain a web-based solution rather than a mobile app. When it comes to administration of the digital solution, one could use already established platforms such as 1177, UMO.se, or, if it is a research-funded project, include university students as administrators. A solution to reach the children in need according to the RG is to provide information on digital platforms where children are active and in everyday settings (eg, on digital boards in buses and schools, social media platforms—TikTok, Discord, etc).

### Dilemma on Using Apps or Other Types of Digital Solutions

An app is a good solution if the purpose is behavioral change or if we want the target group to use the solution on a regular basis. Another advantage of an app is the function of notifications. Having an app on your mobile phone might also create a sense of having a “buddy” or “companion” with you.

On the other hand, if the goal is only conveying information, there are advantages to creating simpler web solutions. It is more cost-effective to develop and maintain web solutions. Other aspects that the RG mentioned were that it is easier to protect the child’s privacy when using web solutions and that there is no need to download and install an app (ie, fewer barriers, age-related restrictions, parental control, and technical issues); hence, web solutions have a lower threshold, which might affect overall accessibility. In addition, it is worth considering if young people really want another mobile app.

According to the RG, the possibility of having both options or even only having a web solution that looks like an app is a worthwhile solution to investigate further. The RG highlights that accessibility should be a decisive factor. In addition, according to the RG, it is important to ask oneself: “Why do you need an app? What should it add or contribute to? What should the user know, feel, or do after using it?”

## Discussion

### Principal Findings

The purpose of this research project was to contribute to equitable and sustainable preventive digital health interventions for COPMI, with the aims of mapping and assessing the quality of available mHealth apps relevant to COPMI; understanding the existing needs for digital health solutions (particularly mHealth) by consulting and collaborating with an interdisciplinary RG (academic researchers, child rights organizations, and IT professionals); and exploring the prerequisites for development, evaluation, implementation, sustainable access, and future digital solutions for COPMI.

In sum, only 3 apps (out of 9) received high scores across the MARS dimensions of functionality, aesthetics, information, and engagement in the expert heuristic evaluation using recorded demonstrations of the apps. This suggests that relatively few of the apps evaluated demonstrated high overall quality according to the assessment criteria used in this study. We also found that the RG preferred apps that were not targeted to COPMI, as these could promote self-identification and reduce stigmatization. Regarding the third aim, the results showed that it was important to find an app that protected users’ privacy by allowing anonymous access to digital support and that mobile apps should be complemented (or replaced) by web-based applications to improve accessibility for children who may not be allowed to download apps without parental permission. Each aim will be discussed in more detail below.

### Mapping and Interdisciplinary Quality Assessment of Internationally Available Apps

A total of 35 apps were initially identified as free and highly ranked apps in English or Swedish. Nine of these apps were selected for MARS evaluation. The results showed Safe Place as the highest-ranked app, with a MARS score of 4.0, followed by the shared runners-up Smiling Mind: Mental Wellbeing and Insight Timer: Meditation App, both scoring 3.8. Thus, these 3 apps emerged as the strongest performers in terms of overall quality in the recording-based MARS assessment. However, the MARS findings should be interpreted in light of the study design. Because the evaluators assessed narrated screen recordings rather than interacting directly with the apps, the scores reflect expert judgment of interface quality, content, and demonstrated functionality.

Overall, the heuristic MARS evaluations showed a consistent pattern across the assessed apps: the dimension of functionality emerged as the top-performing dimension, with an average score of 3.6, followed closely by information provision and aesthetics, with scores of 3.5, and finally, engagement, with a score of 3.1. Interestingly, this is a pattern consistent with other studies by Vaezipour et al [32], O’Connor et al [33], and Svensson et al [34], in which functionality consistently received the highest scores (ie, scores of 4.3, 3.25, and 4.18, respectively), while engagement consistently ranked low (ie, 3.3, 2.12, and 3.04, respectively).

In this study, functionality was the strongest dimension within the recording-based MARS evaluation, reflecting the evaluators’ perceptions of stable performance and intuitive navigation based on the demonstrated app features. In contrast, subjective perceptions of quality were notably lower, suggesting limited willingness among evaluators to recommend or regularly use many of the apps. Perceived engagement potential emerged as another weaker area; evaluators identified several apps as lacking features that could support sustained user interest or interaction, which is also an area that has been pointed out as a significant pitfall among health apps [14,35]. Aesthetics, however, tended to score above average, indicating generally sound visual design across the apps. Information quality was also acceptable for most apps, although the depth, clarity, and tailoring of content varied. Finally, evidence of scientific validation was limited across the set, with only 2 apps supported by easily findable and accessible research. This highlights an ongoing gap between app availability and empirical backing for their effectiveness. This has also been shown in previous research [18,19].

During the MARS evaluation at WS2, both RT and RG highlighted user-friendliness as central to app quality. Apps were rated higher when navigation was intuitive, the design guided users clearly, and the tone felt supportive. Positively perceived apps were aesthetically appealing, calming, pedagogical, and offered an appropriate amount of content and exercises, ideally with some personalization and suitability for varied user groups. Negative feedback focused on unclear or confusing navigation, excessive text, limited content or exercise variety, and inconsistencies such as different narrators. Negative feedback was also raised when there were paywalls within the app, for example, for specific features or content, or when the apps were unclear about their intended audience, offered irrelevant content, or required adult framing. On the other hand, previous research shows that professional guidance when using digital health solutions might improve user engagement [35]. Overall, the findings underscore the importance of clarity, structure, emotional tone, and accessibility in mental health apps for young users.

The quantitative and qualitative findings were largely complementary. While engagement emerged as one of the lowest-rated dimensions in the heuristic MARS assessment, the discussions helped explain these ratings. According to the RG, apps were perceived as less engaging when they contained excessive text, limited opportunities for interaction, few exercises, unclear target audiences, or required adult support to become meaningful. In contrast, highly rated apps were characterized by intuitive navigation, personalization, supportive features, and relevant content. Similarly, the relatively low subjective quality scores may reflect that several apps, despite adequate functionality and aesthetics, did not fully address the needs of COPMI identified by the RG, for example, anonymity, tailored support, peer connection, and easy access to help. Together, these findings suggest that technical quality alone may be insufficient to promote sustained use. The integrated findings further indicate that lower engagement ratings may not only reflect design shortcomings but also a mismatch between existing mHealth apps and the psychosocial needs that the experts in the RG identified as important for COPMI.

The app Smiling Mind: Mental Wellbeing was highly ranked in our study. However, a previous study showed challenges related to engagement, with half of the participants never using the app again after 10 days [36]. The need for improved engagement has been highlighted in a state-of-the-art article in the field of digital mental health care [35]. The authors suggest addressing engagement through personalized features, integration into daily life, strong data protection, human support, just-in-time adaptive interventions, and increased digital literacy among users.

The quality assessment with MARS focused on the above-mentioned dimensions, reflecting the general quality of the evaluated apps. However, the workshop discussions highlighted privacy and data security as particularly important considerations for COPMI. These dimensions are not captured by MARS and should therefore be considered alongside app quality ratings when evaluating suitability for this population.

### Needs Assessment via Consultation With the RG

The results from the needs assessment, based on discussions with the RG, underscore the complexity of support needs and design considerations that professionals and experts perceive as important for COPMI and future mHealth development. These findings also provide important context for interpreting the MARS results, particularly the lower ratings for engagement and subjective quality. Central among the needs identified by the RG were anonymity, low-threshold access, and psychosocial support that can be tailored to individual circumstances. The findings align with concerns about barriers to help-seeking among COPMI [10] and emphasize the suitability of digital solutions as a complement to existing support. Digital solutions that provide opportunities for peer recognition (eg, chatting forums and sharing stories) could be vital for normalizing experiences and reducing potential shame among the target group.

The results also highlight the unmet needs related to knowledge and strategies, including tailored information about parental mental illness, children’s rights, coping and action strategies, and practical guidance for everyday life. Notably, the lack of accessible information about parents’ care and children’s potential involvement in that care was highlighted as a specific gap, which is in line with findings from previous research [13]. Addressing this gap may contribute to reducing shame, increasing transparency, and strengthening trust within family relationships.

A central aspect is the availability of digital platforms for COPMI. Children need to be able to access them without parents’ permission, for example, by downloading apps to their mobile phones. Parents may not want their children to download an app with COPMI-related content, in contrast to parents who have children who have lost a parent and may be in need of an app related to grief [37].

One central finding was that many young people do not identify themselves as belonging to the COPMI group, which may delay or prevent access to support. However, digital solutions do not need to be targeted solely to COPMI, as this may add to further stigmatization and increase privacy risks. A broader reach may facilitate self-identification among COPMI, thus increasing opportunities for young people to recognize their situation and seek support. However, a broader-themed digital solution (eg, improving mental health) could still have some specific content targeted to COPMI, potentially supporting self-identification and increasing awareness of their rights and available support, which in turn could increase knowledge among these children about their rights as COPMI. Another approach to having an app that meets the complex needs of COPMI might be to have a “toolbox approach”; hence, multiple apps could be used instead of having “the” one app. This would also address the potential problem of parental consent for downloading an app with COPMI-specific content.

The results also suggest that mHealth tools should not only provide information but also actively enable relational support, opportunities for support network development, and further help-seeking. Furthermore, the RG stressed that young people should be asked whether they want support and that they should be listened to, reinforcing the importance of participatory and youth-centered design in (developing) digital solutions [38]. Using co-design principles with users has also been suggested as a means of improving engagement [35].

Finally, these findings underscore that accessibility alone is insufficient unless it is accompanied by quality assurance, appropriate timing, and rigorous data security and privacy protection. Privacy and confidentiality breaches have been identified as possible pitfalls in a previous review [14] about mHealth apps in general, and these issues can be regarded as especially important for COPMI. COPMI are a vulnerable group for whom a data security failure is a safety risk. Given that COPMI often lack equitable access to support [8], well-designed mHealth solutions may contribute to widening access to support, particularly in settings where traditional services are difficult to access.

### Exploration of the Prerequisites for Development, Evaluation, Implementation, Sustainable Access, and Further Development

The third aim of this study concerned the prerequisites for ensuring long-term development, implementation, and accessibility to high-quality digital solutions to support COPMI. The findings provide important insights that may inform future work on sustainable digital support for this group.

First, a central challenge is reaching the children who are COPMI and in need of support, regardless of whether they identify themselves as COPMI. Given that both children and parents may be hesitant to acknowledge parental mental illness [10] or its impact on the child and their well-being, digital solutions explicitly targeting COPMI may be perceived as less acceptable than broader mental health support solutions, particularly among children who do not self-identify as belonging to the COPMI group. In addition, equitable access to support must be ensured irrespective of geographical location within the country.

Second, digital solutions must be characterized by high quality and sustainable accessibility over time. Time-limited projects in which digital solutions are developed and occasionally evaluated, but not continuously maintained, updated, and implemented, have limited long-term value. Some digital solutions rely on user fees, either for full or partial access, as a means of financing; however, such models are inappropriate when the intended users are children and adolescents. Sustainable digital solutions require stable, long-term funding that covers both content development and ongoing technical maintenance. Public health care and social services are organized at the regional or municipal level in Sweden, with no clear responsibility for national digital solutions. Representatives from child rights organizations in the RG, operating at a national level, noted that public society often relies on civil society organizations when it comes to long-term responsibility for nationwide support; however, such organizations commonly face insecure and short-term funding. Taken together, these findings highlight the need for robust and long-term funding structures to avoid fragmented and short-lived solutions.

Third, children, as well as professionals who support them, need to be informed about available digital solutions and how to access them. Even high-quality digital tools have limited impact if they are not known to their intended users or to professionals who may support them at local or regional levels. One suggestion discussed within the project was to integrate COPMI-related content into existing, well-established, and trusted digital platforms. This approach aligns with the notion that more general digital solutions may be more acceptable to children who do not (yet) identify themselves as COPMI. Furthermore, recurring dissemination of information about digital solutions for COPMI is necessary and should take place on appropriate platforms. As with development and maintenance, this requires continuous efforts and sustained funding.

### Limitations

Some limitations should be acknowledged as part of the interpretation of this study’s findings. An important methodological limitation is that the MARS evaluations were conducted using narrated screen recordings rather than direct interaction with the apps. Although this approach enabled a larger number of evaluators to assess the apps efficiently due to limited resources and time commitment, it does not fully capture real-world user experience. Consequently, dimensions such as functionality and engagement, as well as subjective quality, should be interpreted with caution, as evaluators assessed engagement potential, interface characteristics, content, and demonstrated functionality rather than direct user experiences. Therefore, the MARS results should be understood as expert heuristic evaluations of app interfaces, content, and demonstrated features rather than measures of actual user experience. Also, MARS does not assess privacy or security, and the rankings reflect general quality rather than data-protection suitability. Privacy and data security were discussed during the workshops and emerged as important considerations for COPMI. Therefore, a quality assessment that relies solely on MARS does not provide a complete assessment of app suitability for this population. Future studies should employ standardized privacy assessment tools to systematically evaluate app permissions, privacy policy availability and transparency, and third-party data-sharing practices in digital health solutions targeting vulnerable youth.

The RT and RG would have liked to have had the possibility to add free-text responses as part of each of the dimensions of the MARS questionnaire, that is, to give a brief explanation for certain ratings. This might have provided us with a deeper understanding of how the quality of the apps is perceived and, consequently, which aspects are most central to the design and features of health apps. To complement the MARS evaluations, we discussed the findings during the workshops, which provided qualitative insights into how participants interpreted app quality, engagement, and relevance. These discussions added contextual understanding but do not remove the limitations associated with the recording-based MARS assessment.

However, we dealt with this limitation regarding the MARS evaluations by using method triangulation strategies, as we had dedicated workshops to further discuss the results of the evaluations and perspectives on each of the apps. As part of the workshops, we had more in-depth conversations, allowing us to discuss the rationale for our app ratings across the MARS dimensions and more freely articulate our perceptions about every app, such as the ones that stood out and the ones deemed inadequate.

### Conclusions

The conclusions are that aesthetics and perceived functionality were generally rated adequate in the recording-based expert evaluation, whereas perceived engagement potential emerged as a weakness. Knowing the causes of low engagement is crucial [35] and, hence, of importance for future research. The integrated findings suggest that technical quality alone is insufficient for sustained engagement. Apps with higher ratings were characterized by personalization, intuitive navigation, and relevant content, while lower-rated apps often lacked interactivity and alignment with the needs identified by the RG. Future research should complement expert heuristic evaluations with hands-on usability testing and evaluations involving COPMI, while also incorporating assessments of privacy and data security [35]. To address the engagement and deployment issues, it is important to actively involve children through participatory and cocreational approaches when designing and developing digital health solutions. Also, future evaluations should include privacy and data security assessments, such as app permissions, privacy policy presence, and third-party sharing.

Effective digital support for COPMI must prioritize anonymity, low-threshold access, strong data protection, and flexibility in delivery. Importantly, digital solutions do not necessarily need to be explicitly targeted to COPMI. Broader mental health solutions that include COPMI-relevant content may represent a less stigmatizing and more accessible pathway to support for young people who do not self-identify as belonging to the group.

Finally, the sustainability of digital solutions (web applications or mobile apps) for COPMI is the biggest challenge for future developments. Long-term impact requires stable funding, continuous maintenance, and partnering with established organizations that already possess the infrastructure, expertise, and reach necessary to provide ongoing support. Our study offers insights into how future digital support for COPMI can be designed, implemented, and sustained, while highlighting the importance of engagement, privacy, accessibility, and long-term organizational commitment.

## Data Availability

The datasets generated or analyzed during this study are available in the GitHub repository [31].

## Abbreviations

COPMI: Children of parents with mental health illness
HSL: Swedish Health and Medical Services Act
MARS: Mobile App Rating Scale
mHealth: mobile health
RG: reference group
RT: research team
WS1: first workshop
WS2: second workshop
WS3: third workshop
